# Use of Dieselized Farm Equipment and Incident Lung Cancer: Findings from the Agricultural Health Study Cohort

**DOI:** 10.1289/ehp.1409238

**Published:** 2015-10-09

**Authors:** Séverine Tual, Debra T. Silverman, Stella Koutros, Aaron Blair, Dale P. Sandler, Pierre Lebailly, Gabriella Andreotti, Jane A. Hoppin, Laura E. Beane Freeman

**Affiliations:** 1 INSERM, UMR 1086 Cancers et Préventions, Caen, France; 2 Université de Caen, Normandie, Caen, France; 3 Centre de Lutte Contre le Cancer François Baclesse, Caen, France; 4 Division of Cancer Epidemiology and Genetics, Occupational and Environmental Epidemiology Branch, National Cancer Institute, National Institutes of Health (NIH), Department of Health and Human Services (DHHS), Rockville, Maryland, USA; 5 Epidemiology Branch, National Institute of Environmental Health Sciences, NIH, DHHS, Research Triangle, North Carolina, USA; 6 Department of Biological Sciences, North Carolina State University, Raleigh, North Carolina, USA

## Abstract

**Background:**

Diesel exhaust is a known lung carcinogen. Farmers use a variety of dieselized equipment and thus may be at increased risk of lung cancer, but farm exposures such as endotoxins may also be protective for lung cancer.

**Objectives:**

We evaluated the relative risk of incident lung cancer, including histological subtype, from enrollment (1993–1997) to 2010–2011 in relation to farm equipment use in the Agricultural Health Study (AHS), a prospective cohort study of pesticide applicators and spouses in Iowa and North Carolina, USA.

**Methods:**

Farm equipment use was reported by 21,273 farmers and 29,840 spouses. Rate ratios (RRs) were estimated separately for farmers and spouses with Poisson regression models adjusted for smoking and other confounders. We conducted stratified analyses by exposure to animals or stored grain, a surrogate for endotoxin exposure.

**Results:**

Daily diesel tractor use (vs. no use) was positively associated with lung cancer in farmers (RR = 1.48; 95% CI: 0.87, 2.50; 35 exposed, 32 unexposed cases), particularly adenocarcinoma (RR = 3.39; 95% CI: 1.23, 9.33; 12 exposed, 7 unexposed cases). The association of adenocarcinoma with daily (vs. low/no) use of diesel tractors was stronger for farmers with no animal or stored grain exposures (RR = 6.23; 95% CI: 2.25, 17.25; 5 exposed, 18 unexposed cases) than among farmers with these exposures (RR = 1.19; 95% CI: 0.51, 2.79; 7 exposed, 27 unexposed cases) (p-interaction = 0.05).

**Conclusions:**

This study provides preliminary evidence of an increased risk of lung adenocarcinoma among daily drivers of diesel tractors and suggests that exposure to endotoxins may modify the impact of diesel exposure on lung cancer risk. Confirmation of these findings with more exposed cases and more detailed exposure information is warranted.

**Citation:**

Tual S, Silverman DT, Koutros S, Blair A, Sandler DP, Lebailly P, Andreotti G, Hoppin JA, Beane Freeman LE. 2016. Use of dieselized farm equipment and incident lung cancer: findings from the Agricultural Health Study Cohort. Environ Health Perspect 124:611–618; http://dx.doi.org/10.1289/ehp.1409238

## Introduction

Diesel exhaust has recently been classified by the International Agency for Research on Cancer as a lung carcinogen based primarily on epidemiological findings in miners and truck drivers and bioassays (Benbrahim-Tallaa et al. 2012). However, other occupational groups have exposure to diesel exhaust (Pronk et al. 2009). For example, farmers and agricultural workers have used diesel-powered equipment, such as tractors, combines, large trucks, and other heavy equipment since at least the 1970s in the United States (Coble et al. 2002; U.S. Census of Agriculture 2015).

Despite their potential exposure to diesel exhaust, it has been observed in multiple studies, including the Agricultural Health Study (AHS), that farmers have lower rates of lung cancer than the general population (Blair et al. 1992; Koutros et al. 2010). This may be explained partially by a lower prevalence of smoking (Blair and Freeman 2009). However, it may also be attributable to endotoxins, a component of the outer membrane of Gram-negative bacteria present in organic dust, which have been linked to reduced risk of lung cancer, likely through immunologic mechanisms (Lundin and Checkoway 2009). High levels of endotoxins have been reported in agricultural settings, particularly in animal farming and during machine harvest (Liebers et al. 2006). A few epidemiologic studies, including the AHS, have shown reduced risks of lung cancer associated with contact with farm animals (dairy farming, poultry, and large numbers of livestock) after adjustment for smoking (Beane Freeman et al. 2012; Mastrangelo et al. 2005).

We evaluated whether the use of dieselized farm equipment was associated with total and subtypes of lung cancer in farmers and their spouses in the AHS, while considering smoking and concomitant potential exposure to endotoxins.

## Methods

*Cohort enrollment and follow-up.* The AHS is a prospective cohort study that includes licensed pesticide applicators (private and commercial applicators) and their spouses in Iowa and North Carolina (Alavanja et al. 1996). Because we were interested in farm-related exposures, we restricted the present analysis to private applicators (i.e., farmers) and their spouses. Farmers were recruited between December 1993 and December 1997 from pesticide certification sessions (84% of eligible farmers enrolled) and completed a self-administered questionnaire during the session. They were given a second, more detailed questionnaire on other occupational exposures to complete at home and return by mail (take-home questionnaire). This questionnaire was completed by 22,916 farmers (44% of 52,394 enrolled farmers) at enrollment. Farmers who returned the take-home questionnaire were similar to nonresponders with regard to demographic characteristics, farming practices, and medical history (Tarone et al. 1997). Spouses of the 43,692 enrolled farmers who reported their marital status as married or living as married were asked to complete a questionnaire, brought home by the farmers, which was different from the farmer take-home questionnaire. A total of 32,345 spouses returned the questionnaire, which we estimated to be 74% of the eligible population.

Cohort members are matched to cancer registry files in Iowa (http://www.public-health.uiowa.edu/shri/) and North Carolina (http://www.schs.state.nc.us/units/ccr/) for case identification and to the state death registries (Iowa: http://www.idph.iowa.gov/health-statistics/vital-records; North Carolina: http://vitalrecords.nc.gov/) and the National Death Index to ascertain vital status (http://www.cdc.gov/nchs/ndi.htm). We identified incident cancers between date of enrollment and 31 December 2010 for North Carolina and 31 December 2011 for Iowa. Histological subtype was coded according to the *International Classification of Diseases for Oncology, 2nd* and *3rd Revision*. We identified cohort members no longer residing in Iowa or North Carolina by linkage to several national databases, including Internal Revenue Service records and address databases, and pesticide license registries of the state agricultural departments. We censored person-time in the year that they left the state. Individuals were followed from enrollment until the earliest of any cancer diagnosis, death, date they left the state, or end of follow-up. The mean time of follow-up was 14.6 years (± 3.8 years) for farmers and 14.5 years (± 3.3 years) for spouses. All participants provided informed consent, and all relevant institutional review boards reviewed and approved the study protocol.

*Exposure assessment.* Information on the use of motorized farm equipment (diesel tractors, gasoline tractors, trucks, and combines or other crop harvesters), including frequency of use during the summer growing season and the winter (nongrowing) season, was collected on the farmer take-home questionnaire and spouse questionnaire. The use of diesel tractors was classified separately from gasoline powered tractors, but questions about trucks and combines or other crop harvesters did not distinguish between fuel types. For farmers, the use of tractors and trucks was categorized into mutually exclusive groups as follows: never or < once/month, 1–3 times a month (monthly) during the summer and/or winter, 1–3 times a week (weekly) during the summer and/or the winter, 6–7 times a week (daily) during only one season, and daily during both seasons. In some cases these categories were collapsed to three levels of exposure: no or low (< 6 days a week during any season), intermediate (≥ 6 days a week during one season only), and high (≥ 6 days a week during both seasons). For spouses, exposure categories for driving diesel tractors, gasoline tractors, and trucks (any fuel) were, respectively: none/low (never or < once a month during any season), and monthly (at least once a month during at least one season). Use of combines or other crop harvesters (any fuel) by farmers was queried according to the number of days of driving during the last summer growing season (never, 1–10, 11–30, 31–100, and > 100 days) and categorized as never, 1–10, 11–30, and ≥ 31 days for the present analyses to ensure at least 5 cases per exposure group. Spouses were asked if they had driven combines or other crop harvesters during the last growing season, and classified as no use, or any use, accordingly.

Among farmers, potential exposure to endotoxins was classified based on self-reported exposure to stored grain and animals. Specifically, farmers were classified as exposed to stored grain if they indicated that they were exposed to stored grain at least once per year, and were classified as unexposed otherwise. Farmers were classified as exposed to farm animals if they indicated that their major source of income was beef cattle, dairy cattle, hogs/swine, poultry, sheep, eggs, or other farm animals, or if they reported any livestock or poultry on their farm the previous year; otherwise they were classified as unexposed to farm animals. Finally, we created a three-category variable to capture potential exposure to endotoxins from grains or animals: no animal or grain exposure, exposure to animals or stored grain but not both, exposure to both animals and stored grain. Spouses were classified as having potential exposure to endotoxins if they indicated that they had direct contact with dairy cattle, beef cattle, swine/hogs, poultry, or sheep at least once a year during the previous 12 months; otherwise they were classified as unexposed. Spouses were not asked about exposure to stored grain.

*Statistical analysis.* Only farmers who completed the take-home questionnaire were included in this analysis (*n* = 22,916). We excluded individuals with prevalent cancers (i.e., those diagnosed before enrollment; *n* = 599) and those who were not living in Iowa or North Carolina at enrollment (*n* = 83). Due to a low number of exposed cases (< 5 exposed cases), smokers of tobacco products other than cigarettes (pipes, cigars, or cigarillos) (*n* = 396) and individuals with missing values for race (*n* = 517) or missing information for potential endotoxin-related exposures (*n* = 48) were additionally excluded, leaving 21,273 farmers (92.8% of the responders to the take-home questionnaire, mainly males: 97.5%). We included only female spouses of the 43,692 enrolled farmers (32,125 females, 99.3% of spouses). Similar exclusion criteria as for farmers were used for the spouses: prevalent cancers (*n* = 907); spouses who were not living in Iowa or North Carolina at enrollment (*n* = 110); smokers of pipes, cigars, or cigarillos (*n* = 11); and missing information for potential endotoxin-related exposures (*n* = 1,257), leaving 29,840 female spouses (92.3%).

We fit Poisson regression models to estimate rate ratios (RR) and 95% confidence intervals (CI), associated with driving different types of farm equipment, using the logarithm of person-time as an offset term in the GENMOD procedure (SAS version 9.2; SAS Institute Inc.). We evaluated associations among farmers and spouses separately, and by histological subtypes when there were at least 50 cases overall and at least 5 exposed cases in the exposure category (for farmers: adenocarcinomas, squamous cell carcinomas, and small cell carcinomas; and for spouses: adenocarcinomas) (Table 1). For farmers, rate ratios were adjusted for age at enrollment (< 55, 55–59, 60–64, 65–69, 70–74, ≥ 75 years), pack-years of cigarette smoking (nonsmokers, < 20, 20–39, 40–59, ≥ 60, unknown), level of education (beyond high school, high school, above high school, unknown), state (North Carolina, Iowa), race (white, other than white), and potential exposure to endotoxins (no animals or grain exposure, exposure to animals or stored grain but not both, exposure to both animals and stored grain). For spouses, rate ratios were adjusted for age at enrollment (< 55, 55–59, 60–64, 65–69, 70–74, ≥ 75 years), pack-years of cigarette smoking (nonsmokers, < 20, 20–39, ≥ 40, unknown), level of education (< high school, high school, > high school, unknown), and exposure to farm animals in the year before enrollment (yes/no). All these potential confounders were collected by questionnaire.

**Table 1:** General characteristics of farmers and female spouses of farmers in the Agricultural Health Study, by lung cancer status [*n* (%)].

Characteristics	Farmers		Female spouses of farmers	
	Cases (*n *= 281)	Noncases (*n *= 20,992)	Cases (*n *= 165)	Noncases (*n *= 29,675)
Age at enrollment (years)
< 55	70 (25)	13,615 (65)	71 (43)	21,714 (73)
55–59	51 (18)	2,451 (12)	26 (16)	3,298 (11)
60–64	66 (24)	2,127 (10)	31 (19)	2,370 (8)
65–69	49 (17)	1,554 (7)	19 (11)	1,402 (5)
70–74	32 (11)	832 (4)	13 (8)	645 (2)
≥ 75	13 (5)	413 (2)	5 (3)	246 (1)
Pack-years of cigarette smoking^*a*^
Nonsmokers	30 (11)	11,148 (53)	54 (33)	20,929 (71)
< 20	55 (20)	6,043 (29)	35 (21)	6,197 (21)
20–39	74 (26)	1,884 (9)	53 (32)	1,163 (4)
40–59	35 (12)	619 (3)	18 (11)	386 (1)
≥ 60	56 (20)	551 (3)
Missing	31 (11)	747 (3)	5 (3)	1,000 (3)
State
Iowa	115 (41)	13,825 (66)	93 (56)	20,320 (68)
North Carolina	166 (59)	7,167 (34)	72 (44)	9,355 (32)
Race
White	265 (94)	20,611 (98)	163 (99)	29,138 (98)
Other than white	16 (6)	381 (2)	2 (1)	498 (2)
Missing	NA	NA	0 (0)	39 (0)
Level of education
Less than high school	35 (13)	920 (4)	8 (5)	447 (2)
High school	147 (52)	10,845 (52)	86 (52)	11,588 (39)
Above high school	77 (27)	8,737 (42)	64 (39)	17,297 (58)
Missing	22 (8)	490 (2)	7 (4)	343 (1)
History of lung diseases^*b*^
Never	225 (80)	19,184 (91)	144 (87)	28,071 (95)
Ever	30 (11)	839 (4)	19 (12)	1,295 (4)
Missing	26 (9)	969 (5)	2 (1)	309 (1)
Familial history of lung cancer
Never	220 (78)	18,544 (88)	142 (86)	26,651 (90)
Ever	26 (9)	1,304 (6)	19 (12)	2,513 (8)
Missing	35 (13)	1,144 (6)	4 (2)	511 (2)
Years worked/lived on a farm
< 20	27 (10)	2,159 (10)	25 (15)	8,128 (27)
20–29	27 (10)	2,823 (13)	21 (13)	5,348 (18)
≥ 30	221 (78)	15,628 (75)	118 (71)	15,893 (54)
Missing	6 (2)	382 (2)	1 (1)	306 (1)
Lifetime total pesticide exposure (days)
0–50	50 (18)	3,506 (17)	109 (66)	19,248 (65)
51–100	25 (9)	2,085 (10)	10 (6)	1,782 (6)
101–250	74 (26)	7,169 (34)	15 (9)	2,868 (10)
> 250	96 (34)	7,064 (34)	10 (6)	1,525 (5)
Missing	36 (13)	1,168 (5)	21 (13)	4,252 (14)
Animals and stored grain^*c*^
Neither of these exposures	98 (35)	3,812 (18)	102 (62)	13,320 (45)
At least one of these exposures	183 (65)	17,180 (82)	63 (38)	16,355 (55)
Only one exposure	95 (34)	5,909 (28)	NA
Both exposures	88 (31)	11,271 (54)	NA
Histological subtypes
Adenocarcinoma	78 (28)		69 (42)
Squamous cell carcinoma	75 (26)		20 (12)
Small cell carcinoma	50 (18)		30 (18)
Large cell carcinoma	11 (4)		2 (1)
Other subtypes	67 (24)		44 (27)
NA, Not applicable. ^***a***^For spouses: categories > 40 pack-years were collapsed to fulfill the criteria of a minimum of 5 exposed cases. ^***b***^Self-reported diagnosis of lung diseases (chronic bronchitis or emphysema). ^***c***^No information on handling stored grain was collected for spouses.				

A Wald test was performed to test for trend associated with the frequency of use of farm equipment, treating the categorical variable as ordinal in the model. Because farmers often drive more than one type of farm equipment, we also evaluated cancer risk adjusting for other equipment types. We investigated exposure to endotoxin-related activities as a potential effect modifier of the relation between use of farm equipment and lung cancer risk by adding cross-product terms between the frequency of driving farm equipment and exposure to animals and/or stored grain (yes/no) to the Poisson model and conducting a likelihood ratio test.

In our final models, we included pack-years of smoking; however, we performed sensitivity analyses controlling for other smoking metrics (smoking duration: for both farmers and spouses: < 10, 10–19, 20–29, 30–39, and ≥ 40 years; smoking history: for farmers: never, former smoker < 3.75 pack-years, former smoker 3.75–14.9 pack-years, former smoker ≥ 15 pack-years, current smoker < 11.5 pack-years, current smoker 11.5–28.4 pack-years, current smoker ≥ 28.5 pack-years; for spouses: never, former smoker < 1.25 pack-years, former smoker 1.25–7.4 pack-years, former smoker ≥ 7.5 pack-years, current smoker < 7.5 pack-years, current smoker 7.5–18.74 pack-years, current smoker ≥ 18.75 pack-years). We also performed sensitivity analyses controlling for the lifetime number of days of pesticide use and for specific pesticides that had been previously associated with lung cancer in the AHS (dicamba, metolachlor, pendimethalin, diazinon, dieldrin, carbofuran, and chlorpyrifos) (Alavanja et al. 2004). We also conducted sensitivity analyses excluding subjects with previous history of nonmalignant lung diseases (self-reported diagnosis of chronic bronchitis or emphysema) and with family history of lung cancer in first-degree relatives.

Statistical analyses were performed using SAS version 9.2. We used the P1REL0612 release of the AHS database. All statistical tests were two sided, and a *p*-value < 0.05 was reported as significant.

## Results

Selected characteristics of farmers and spouses are displayed in Table 1. Overall, there were 281 and 165 incident lung cancer cases diagnosed among 21,273 farmers and 29,840 spouses, respectively. Lung cancer cases were older, smoked more frequently, came more frequently from North Carolina, had a lower level of education, and reported more frequently a history of nonmalignant respiratory diseases and a family history of lung cancer than non-cases.

*Overall lung cancer risk.* Among farmers, we observed an increased risk associated with the daily driving of a diesel tractor in both seasons (RR = 1.48; 95% CI: 0.87, 2.50) compared to nonusers of diesel tractors, after adjustment for confounders (Table 2). The use of other farm equipment (gasoline tractors, combines or other types of crop harvesters, and trucks) was not associated with lung cancer among farmers. Among spouses, the use of diesel and gasoline tractors was inversely associated with lung cancer (diesel tractor: RR = 0.71; 95% CI: 0.48, 1.06; gasoline tractor: RR = 0.67; 95% CI: 0.44, 1.04). After mutual adjustment for both types of tractors, association for diesel tractor use remained unchanged for farmers (RR = 1.38; 95% CI: 0.81, 2.37), was attenuated among spouses (RR = 0.88; 95% CI: 0.54, 1.43), and did not change for gasoline tractor use for spouses (RR = 0.67; 95% CI: 0.40, 1.15) (see Supplemental Material, Table S1). The use of other farm equipment (combines or other types of crop harvesters and trucks) was not associated with lung cancer for spouses.

**Table 2:** Associations between driving farm equipment and the overall lung cancer risk among farmers and female spouses of farmers, Agricultural Health Study.

Exposure	Farmers		Spouses of farmers	
	Cases^*a*^ (*n *= 281) (*n*)	RR^*b*^ (95% CI)	Cases^*a*^ (*n *= 165) (*n*)	RR^*b*^ (95% CI)
Diesel tractors^*c*^
No/< monthly	32	1.00	121	1.00
≥ Monthly ≥ 1 season	26	1.19 (0.71, 2.01)	34	0.71 (0.48, 1.06)
Weekly ≥ 1 season	121	1.11 (0.74, 1.68)
Daily in one season	51	1.21 (0.75, 1.95)
Daily in both seasons	35	1.48 (0.87, 2.50)
*p* for trend^*d*^		0.18
Gasoline tractors^*c*^
No/< monthly	91	1.00	128	1.00
≥ Monthly ≥ 1 season	56	0.87 (0.62, 1.22)	27	0.67 (0.44, 1.04)
≥ Weekly ≥ 1 season	90	1.07 (0.79, 1.44)
Daily in one season	12	1.03 (0.56, 1.89)
Daily in both seasons	13	1.20 (0.66, 2.19)
*p* for trend^*d*^		0.49
Combines or other types of crop harvesters^*c*^
No/< monthly	87	1.00	149	1.00
1–10 days	57	1.11 (0.78,1.58)	12	0.74 (0.41, 1.34)
11–30 days	89	1.11 (0.78,1.57)
≥ 31 days	30	0.80 (0.51,1.26)
*p* for trend^*d*^		0.57
Trucks^*c*^
No or < monthly	83	1.00	108	1.00
≥ Monthly ≥ 1 season	41	0.85 (0.58, 1.23)	47	0.82 (0.57, 1.16)
≥ Weekly ≥ 1 season	69	0.89 (0.65, 1.24)
Daily in one season	15	1.01 (0.58, 1.76)
Daily in both seasons	52	0.80 (0.55, 1.15)
*p* for trend^*d*^		0.31
^***a***^Case counts do not sum to total counts because of missing values for exposure variables. ^***b***^For farmers, rate ratios were adjusted for age, pack-years (nonsmokers, < 20, 20–39, 40–59, ≥ 60, missing), state, race, level of education, and current exposure to animals and stored grain (no, one exposure, both exposures); for spouses: rate ratios were adjusted for age, pack-years (nonsmokers, < 20, 20–39, ≥ 40, missing), level of education, and exposure to farm animals in the year before enrollment. ^***c***^The frequency of use of farm equipment in the year was not collected by the spouses’ questionnaire. ^***d***^Test for trend, *p*-value obtained by treating the categorical variable as ordinal.				

*Exposure to animals or stored grain as an effect modifier.* The RR for daily driving of diesel tractors compared with no or low use was higher for farmers not exposed to animals or stored grain (RR = 1.83; 95% CI: 0.79, 4.25; 6 exposed and 72 unexposed cases) than for farmers who did raise animals or handle stored grain (RR = 1.19; 95% CI: 0.78, 1.81; 29 exposed cases and 107 exposed cases), though the difference was not statistically significant (*p*-interaction = 0.63) (Table 3). After adjustment for exposure to gasoline tractors, the RRs were 1.45 (95% CI: 0.55, 3.82) and 1.24 (95% CI: 0.82, 1.88) for daily versus no/low use of diesel tractors among those without and with endotoxin-related exposures, respectively (see Supplemental Material, Table S2). Associations between lung cancer and other types of farm equipment also did not show clear variation according to potential exposure to endotoxins in farmers, though results are difficult to interpret given the small numbers of observations.

**Table 3:** Associations between driving farm equipment and the overall lung cancer risk, by exposure to endotoxin-related activities, among farmers and female spouses of farmers, Agricultural Health Study.

Population/exposure	Nonexposed to endotoxin-related activities^*a*^		Exposed to endotoxin-related activities^*a*^		*p* for interaction^*d*^
	Cases^*b*^ (*n*)	RR^*c*^ (95% CI)	Cases^*b*^(*n*)	RR^*c*^ (95% CI)	
Farmers
Diesel tractors
No/low^*e*^	72	1.00	107	1.00	0.63
Intermediate^*f*^	10	0.99 (0.50, 1.93)	41	1.11 (0.77, 1.61)
High^*g*^	6	1.83 (0.79, 4.25)	29	1.19 (0.78, 1.81)
Gasoline tractors
No/low^*e*^	82	1.00	155	1.00	0.33
Intermediate^*f*^/high^*g*^	6	1.63 (0.71, 3.75)	19	0.99 (0.61, 1.60)
Combines or other types of crop harvesters
0 day	49	1.00	38	1.00	0.43
1–30 days	33	1.38 (0.88, 2.17)	113	0.94 (0.41, 2.18)
≥ 31 days	6	0.80 (0.34, 1.89)	24	0.72 (0.14, 3.76)
Trucks
No/low^*e*^	64	1.00	129	1.00	0.50
Intermediate^*f*^	4	0.70 (0.25, 1.91)	11	1.38 (0.74, 2.56)
High^*g*^	18	0.84 (0.49, 1.41)	34	0.88 (0.59, 1.30)
Spouses
Diesel tractors
No/< monthly	75	1.00	46	1.00	0.01
≥ Monthly	19	1.24 (0.75, 2.07)	15	0.41 (0.23, 0.73)
Gasoline tractors
No/< monthly	82	1.00	46	1.00	0.16
≥ Monthly	13	0.93 (0.52, 1.67)	14	0.51 (0.28, 0.93)
Combines or other types of crop harvesters
0 day	94	1.00	55	1.00	0.66
≥ 1 day	5	0.87 (0.35, 2.13)	7	0.66 (0.30, 1.45)
Trucks
No/< monthly	72	1.00	36	1.00	0.78
≥ Monthly	23	0.86 (0.53, 1.37)	24	0.77 (0.46, 1.30)
^***a***^Endotoxin-related activities were defined by current exposure to animals or stored grain in farmers and exposure to farm animals in the year before enrollment in spouses. ^***b***^Case counts do not sum to total counts because of missing values for exposure variables. ^***c***^For farmers: rate ratios were adjusted for age, cigarette pack-years (non-smokers, < 20, 20–39, 40–59, ≥ 60, missing), state, race, and level of education; for spouses: rate ratios were adjusted for age, pack-years (non-smokers, < 20, 20–39, ≥ 40, missing), and level of education. ^***d***^*p* for interaction was obtained from the likelihood ratio test by adding cross-product terms between each category of exposure and the variable reflecting potential exposure to endotoxins. ^***e***^No or low exposure was defined by driving < daily in one season. ^***f***^Intermediate exposure: daily driving in one season. ^***g***^High exposure: daily driving in both seasons.					

Among spouses, there was an evidence of heterogeneity in risk of lung cancer associated with driving diesel tractors by exposure to animals (Table 3). The use of diesel tractors compared with no use was positively associated with lung cancer for spouses not exposed to animals (RR = 1.24; 95% CI: 0.75, 2.07) and inversely associated for spouses who did raise animals (RR = 0.41; 95% CI: 0.23, 0.73, *p*-interaction = 0.01). Use of gasoline tractor was also inversely associated with lung cancer among spouses exposed to animals (RR = 0.51; 95% CI: 0.28, 0.93), though the difference according to exposure to animals was not statistically significant (*p*-interaction = 0.16). After adjustment for the use of gasoline tractor, the increased risk associated with the use of diesel tractor was strengthened among spouses not exposed to animals (RR = 1.81; 95% CI: 0.98, 3.34) and the inverse association remained unchanged among spouses exposed to animals (RR = 0.42; 95% CI: 0.21, 0.86, *p*-interaction < 0.01) (see Supplemental Material, Table S2). Associations between lung cancer and other types of farm equipment did not show clear variation according to exposure to animals in spouses (Table 3; see also Supplemental Material, Table S2).

*Lung cancer risk by histological subtypes.* A significant linear trend was observed between the frequency of use of diesel tractors and lung adenocarcinomas among farmers (*p*-trend = 0.01) (Table 4). Farmers who drove diesel tractors every day (12 cases) had a higher risk of lung adenocarcinomas than farmers who did not drive diesel tractors (7 cases) (RR = 3.39; 95% CI: 1.23, 9.33). Use of diesel tractors was also positively associated with squamous cell carcinomas without significant linear trend (*p*-trend = 0.95) and was not associated with small cell carcinomas (Table 4). Adjustment for use of gasoline tractor did not substantially change associations between use of diesel tractor and lung histological subtypes (see Supplemental Material, Table S3).

**Table 4:** Associations between driving farm equipment and lung cancer histological subtypes among farmers and female spouses of farmers, Agricultural Health Study.

Exposure	Farmers						Spouses	
	Adenocarcinoma		Squamous cell carcinoma		Small cell carcinoma		Adenocarcinoma	
	Cases^*a*^ (*n *= 78) (*n*)	RR^*b*^ (95% CI)	Cases^*a*^ (*n *= 75) (*n*)	RR^*c*^ (95% CI)	Cases^*a*^ (*n *= 50) (*n*)	RR^*c*^ (95% CI)	Cases^*a*^ (*n *= 69) (*n*)	RR^*d*^ (95% CI)
Diesel tractors
No/< monthly	7	1.00	6	1.00	12	1.00	47	1.00
≥ Monthly ≥ 1 season	7	1.53 (0.53, 4.40)	9	2.16 (0.76, 6.14)			18	1.00 (0.56, 1.76)
≥ Weekly ≥ 1 season	31	1.46 (0.62, 3.45)	36	1.64 (0.66, 4.07)	24	0.98 (0.46, 2.05)
Daily in one season	19	2.41 (0.94, 6.13)	12	1.36 (0.48, 3.85)	5	0.56 (0.19, 1.70)
Daily in both seasons	12	3.39 (1.23, 9.33)	7	1.28 (0.40, 4.13)	7	1.16 (0.41, 3.28)
*p* for trend^*e*^		0.01		0.95		0.87
Gasoline tractors
No/< monthly	24	1.00	25	1.00	20	1.00	49	1.00
≥ Monthly ≥ 1 season	16	0.93 (0.49, 1.77)	16	0.90 (0.48, 1.71)	8	0.59 (0.26, 1.35)	16	1.11 (0.62, 1.99)
≥ Weekly ≥ 1 season	28	1.30 (0.74, 2.29)	23	0.93 (0.52, 1.68)	11	0.61 (0.29, 1.31)
Daily ≥ 1 season	8	1.51 (0.66, 3.46)	5	0.74 (0.27, 1.98)	8	1.65 (0.69, 3.95)
*p* for trend^*e*^		0.21		0.62		0.93
Combines or other types of crop harvesters
Never	26	1.00	18	1.00	15	1.00	60	1.00
1–10 days	11	0.76 (0.36, 1.57)	25	2.27 (1.19, 4.34)	9	1.03 (0.43, 2.48)	8	1.29 (0.61, 2.73)
11–30 days	29	1.26 (0.67, 2.35)	20	1.16 (0.56, 2.39)	17	1.40 (0.63, 3.14)
≥ 31 days	10	1.01 (0.46, 2.23)	8	1.02 (0.42, 2.50)	5	0.79 (0.27, 2.33)
*p* for trend^*e*^		0.63		0.72		0.97
Trucks
No/< monthly	24	1.00	23	1.00	16	1.00	47	1.00
≥ Monthly ≥ 1 season	9	0.66 (0.31, 1.43)	11	0.81 (0.39, 1.68)	9	0.90 (0.40, 2.05)	18	0.74 (0.42, 1.29)
≥ Weekly ≥ 1 season	22	1.05 (0.58, 1.90)	15	0.68 (0.35, 1.32)	13	0.75 (0.35, 1.59)
Daily ≥ 1 season	19	0.94 (0.49, 1.78)	17	0.79 (0.40, 1.54)	10	0.47 (0.21, 1.09)
*p* for trend^*e*^		0.93		0.38		0.08
^***a***^Case counts do not sum to total case counts because of missing values for exposure variables. ^***b***^Adjusted for age (< 55, 55–59, 60–64, 65–69, ≥ 70), pack-years (nonsmokers, < 20, 20–39, 40–59, ≥ 60, missing), state, race, level of education (less than high school, high school, above high school, unknown), and current exposure to animals and stored grain (no, one exposure, both exposures). ^***c***^Adjusted for age (< 55, 55–59, 60–64, 65–69, ≥ 70), pack-years (nonsmokers or < 20, 20–39, 40–59, ≥ 60, missing), state, race, level of education (less than high school, high school, above high school, unknown), and current exposure to animals and stored grain (No, one exposure, both exposures). ^***d***^Adjusted for age (< 55, 55–59, 60–64, 65–69, ≥ 70), pack-years (nonsmokers, < 20, 20–39, ≥ 40, missing), level of education (high school or less, above high school, unknown), and exposure to farm animals in the year before enrollment. ^***e***^Test for trend, *p*-value obtained by treating the categorical variable as ordinal.								

Farmers who daily drove gasoline tractors during at least one season (8 cases) had a higher risk of lung adenocarcinomas than farmers who did not drive these tractors (24 cases) (RR = 1.51; 95% CI: 0.66, 3.46, *p*-trend = 0.21) (Table 4). This association was attenuated after adjustment for diesel tractors (RR = 1.25; 95% CI: 0.54, 2.89, *p*-trend = 0.38) (see Supplemental Material, Table S3). They also had an increased risk for small cell carcinomas (RR = 1.65; 95% CI: 0.69, 3.95; 8 exposed and 20 unexposed cases; *p*-trend = 0.93) (Table 4), strengthened after adjustment for diesel tractors (RR = 1.92; 95% CI: 0.77, 4.77) (see Supplemental Material, Table S3), though the trend was not significant (*p* = 0.81).

Farmers who drove combines or other crop harvesters fewer than 10 days had a higher risk of squamous cell carcinomas than farmers who did not drive this type of equipment (RR = 2.27; 95% CI: 1.19, 4.34, *p*-trend = 0.72). The use of trucks was inversely associated with small cell carcinomas (daily during at least one season: RR = 0.47; 95% CI: 0.21, 1.09, *p*-trend = 0.08) (Table 4).

Among spouses, no association was found between use of any type of farm equipment and lung histological subtypes (Table 4).

*Exposure to animals or stored grain as an effect modifier by histological subtypes.* The association between adenocarcinoma and daily use of diesel tractors in both seasons (vs. less than daily use in one season) was stronger among farmers who were not exposed to animals or stored grain (RR = 6.23; 95% CI: 2.25, 17.25; 5 exposed and 18 unexposed cases) than among farmers who were exposed to animals or stored grain (RR = 1.19; 95% CI: 0.51, 2.79; 7 exposed and 27 unexposed cases; *p*-interaction = 0.05) (Table 5). Association for the highest category of combine use (≥ 31 days) was also stronger among farmers not exposed to animals or stored grain (RR = 2.49; 95% CI: 0.79, 7.91; 4 exposed and 12 unexposed cases) than among those who were exposed to animals or stored grain (RR = 0.47; 95% CI: 0.18, 1.27; 6 exposed and 14 unexposed cases; *p*-interaction = 0.06). Daily use of gasoline tractors in at least one season (vs. less than daily) was associated with an increased risk of lung adenocarcinomas among farmers not exposed to animals or stored grain (RR = 2.95; 95% CI: 0.87, 9.99; 3 exposed and 23 unexposed cases) than among those who were exposed to animals or stored grain (RR = 0.89; 95% CI: 0.35, 2.24; 5 exposed and 45 unexposed cases; *p*-interaction = 0.14).

**Table 5:** Associations between driving farm equipment and risk for lung adenocarcinoma, by exposure to endotoxin-related activities, among farmers and female spouses of farmers, Agricultural Health Study.

Population/exposure	Nonexposed to endotoxin-related activities^*a*^		Exposed to endotoxin-related activities^*a*^		*p* for interaction^*d*^
	Cases^*b*^ (*n*)	RR^*c*^ (95% CI)	Cases^*b*^ (*n*)	RR^*c*^ (95% CI)	
Farmers
Diesel tractors
No/low^*e*^	18	1.00	27	1.00	0.05
Intermediate^*f*^	3	1.35 (0.39, 4.68)	16	1.70 (0.90, 3.21)
High^*g*^	5	6.23 (2.25, 17.25)	7	1.19 (0.51, 2.79)
Gasoline tractors
No/low^*e*^	23	1.00	45	1.00	0.14
Intermediate^*f*^/high^*g*^	3	2.95 (0.87, 9.99)	5	0.89 (0.35, 2.24)
Combines or other types of crop harvesters
0 day	12	1.00	14	1.00	0.06
1–30 days	10	1.67 (0.71, 3.93)	30	0.63 (0.32, 1.23)
≥ 31 days	4	2.49 (0.79, 7.91)	6
Trucks
No/low^*e*^	16	1.00	39	1.00	0.51
Intermediate^*f*^/high^*g*^	8	1.19 (0.51, 2.81)	11	0.83 (0.41, 1.66)
Spouses
Diesel tractors
No/< monthly	30	1.00	17	1.00	0.17
≥ Monthly	9	1.47 (0.69, 3.12)	9	0.68 (0.30, 1.52)
Gasoline tractors
No/< monthly	33	1.00	16	1.00	0.70
≥ Monthly	7	1.24 (0.55, 2.82)	9	0.99 (0.44, 2.25)
Combines or other types of crop harvesters
0 day	39	1.00	21	1.00	0.94
≥ 1 day	3	1.24 (0.38, 4.03)	5	1.32 (0.50, 3.52)
Trucks
No/< monthly	31	1.00	16	1.00	0.80
≥ Monthly	9	0.79 (0.37, 1.68)	9	0.68 (0.30, 1.55)
^***a***^Endotoxin-related activities were defined by current exposure to animals or stored grain in farmers and exposure to farm animals in the year before enrollment in spouses. ^***b***^Case counts do not sum to total counts because of missing values for exposure variables. ^***c***^For farmers: Rate ratios were adjusted for age, pack-years (nonsmokers, < 20, 20–39, 40–59, ≥ 60, missing), state, race, and level of education (less than high school, high school, above high school, unknown); for spouses: rate ratios were adjusted for age, pack-years (nonsmokers, < 20, 20–39, ≥ 40, missing), and level of education (high school or less, above high school, unknown). ^***d***^*p* for interaction was obtained from the likelihood ratio test by adding cross-product terms between each category of exposure and the variable reflecting potential exposure to endotoxins. ^***e***^No or low exposure was defined by a driving < daily in one season. ^***f***^Intermediate exposure: daily driving in one season. ^***g***^High exposure: daily driving in both seasons.					

After mutual adjustment for diesel and gasoline tractors, all increased risks among farmers not exposed to animals or stored grain were attenuated but still elevated (diesel tractor: RR = 4.09; 95% CI: 1.18, 14.18; combines or other crop harvesters: RR = 1.97; 95% CI: 0.54, 7.16; gasoline tractors: RR = 1.79; 95% CI: 0.45, 7.16) and associations remained unchanged among farmers exposed to animals or stored grain (diesel tractor: RR = 1.23; 95% CI: 0.52, 2.88; *p*-interaction = 0.24; combines or other crop harvesters: RR = 0.37; 95% CI: 0.14, 1.02; *p*-interaction = 0.09; gasoline tractors: RR = 0.79; 95% CI: 0.31, 2.04; *p*-interaction = 0.35) (see Supplemental Material, Table S4).

Among spouses, exposure to animals did not significantly modify the association between farm equipment use and lung adenocarcinomas (Table 5). However, after mutual adjustment for diesel and gasoline tractors, the RR for driving of diesel tractors compared with no use was higher for spouses not exposed to animals (RR = 2.16; 95% CI: 0.88, 5.33; 9 exposed and 29 unexposed cases) than for spouses who did raise animals (RR = 0.47; 95% CI: 0.17, 1.32; 8 exposed cases and 17 exposed cases, *p*-interaction = 0.03) (see Supplemental Material, Table S4). These associations need however to be interpreted with caution considering the small number of cases.

We estimated associations using other smoking metrics. Associations between daily use of diesel tractors (vs. no use) and lung cancer did not change substantially after adjustment for smoking duration (RR = 1.36; 95% CI: 0.80, 2.30) or after adjustment for smoking history (pack-years among former smokers and pack-years among current smokers) (RR = 1.46; 95% CI: 0.86, 2.49). We saw no evidence of confounding by smoking between daily use of diesel tractor and lung adenocarcinomas (adjusted for smoking duration: RR = 3.09; 95% CI: 1.12, 8.54, *p*-trend = 0.01; adjusted for smoking history: RR = 3.46; 95% CI: 1.25, 9.55, *p*-trend < 0.01). Association between lung cancer risk and daily use of diesel tractor was also similar to those reported in Table 3 among farmers not exposed to animals or stored grain (adjusted for smoking duration: RR = 1.81; 95% CI: 0.78, 4.21; adjusted for smoking history: RR = 2.12; 95% CI: 0.91, 4.91) and among farmers exposed to animals or stored grain (adjusted for smoking duration: RR = 1.18; 95% CI: 0.78, 1.80, *p*-interaction = 0.60; adjusted for smoking history: RR = 1.22; 95% CI: 0.80, 1.85, *p*-interaction = 0.50). Point estimates did not change substantially from those reported in Table 5 for adenocarcinoma among farmers not exposed to animals or stored grain (adjusted for smoking duration: RR = 6.20; 95% CI: 2.23, 17.24; adjusted for smoking history: RR = 6.86; 95% CI: 2.47, 19.02) and among farmers exposed to animals or stored grain (adjusted for smoking duration: RR = 1.21; 95% CI: 0.52, 2.84, *p*-interaction = 0.04; adjusted for smoking history: RR = 1.22; 95% CI: 0.52, 2.86, *p*-interaction = 0.04).

For all these associations, we saw no evidence of confounding by use of pesticides (overall use and use of pesticides previously associated with lung cancer risk) (data not shown). We also performed analyses excluding participants with a history of lung cancer in first-degree relatives (*n* = 1,330 farmers and 2,532 spouses) and those with a history of chronic respiratory disease (*n* = 869 farmers and 1,314 spouses) with no change in estimates higher than 20% (data not shown).

## Discussion

In this prospective study, we observed an increased risk of lung cancer overall, especially for adenocarcinoma, associated with the highest frequency of driving diesel tractors among farmers. We also reported positive associations with driving diesel tractors among farmers not exposed to animals or stored grain for adenocarcinoma and among spouses not exposed to animals for lung cancer and adenocarcinomas, after adjustment for use of gasoline tractors. These findings should, however, be interpreted with caution considering crude markers of exposure of both diesel engine exhaust and endotoxins and small number of cases in analyses stratified by exposure to animals and stored grain, and by histological subtypes. To our knowledge, this is the first prospective study to examine the role of diesel exhaust exposure on lung cancer risk in agriculture, and to consider the potentially modifying role of agricultural exposures such as endotoxins that may be associated with a decreased risk of lung cancer.

Increased risks of lung cancer have been estimated among workers highly exposed to diesel exhaust, such as underground miners, in the highest cumulative exposure category (≥ 878 μg/m^3^–year), based on quantitative estimates of respirable elemental carbon [odds ratio (OR) = 5.10; 95% CI: 1.88, 13.87] (Silverman et al. 2012). An increased risk was also reported in the trucking industry (OR = 2.77; 95% CI: 0.85, 9.00 per 1,000 μg/m^3^–year) (Garshick et al. 2012). Although no measurement data on engine exhaust have been reported in agricultural settings (Pronk et al. 2009), farming has been usually classified as a low diesel-exposed setting compared with other occupationally exposed groups (Olsson et al. 2011). Therefore, the results of the present analysis, although based on the frequency of current driving of diesel tractors, are supportive of the results in the more highly exposed groups and consistent with other analyses with light to moderate levels of exposure which reported positive associations for lung cancer overall (estimated risks from 1.3 to 1.5) (Bhatia et al. 1998; Lipsett and Campleman 1999; Olsson et al. 2011). Few epidemiologic studies have investigated the role of driving farm equipment specifically on lung cancer risk. A pooled case–control analysis in Germany did not show any strong association with full-time driving of tractors by farmers, using job title (OR = 1.29; 95% CI: 0.78, 2.14). Association with the highest duration of time of employment was, however, significantly positive (> 30 years: OR = 6.81; 95% CI: 1.17, 39.51), but these results need to be interpreted with caution due to the low precision. No information was available for the type of tractor (diesel or gasoline) (Brüske-Hohlfeld et al. 1999).

Although based on a small number of cases, our results suggest that diesel exhaust exposure may be more strongly associated with adenocarcinoma than other histological types among AHS farmers. This finding seems to be consistent with results of meta-analyses and a few recent studies indicating that adenocarcinoma is the type most strongly associated with PM_2.5_ and PM_10_ (particulate matter with diameters ≤ 2.5 and ≤ 10 μm) components of diesel exhaust (Hamra et al. 2014; Puett et al. 2014; Raaschou-Nielsen et al. 2013). To our knowledge, only one case–control study estimated associations between driving farm equipment based on job title and the histological type. De Stefani et al. (2005) reported a significant association with lung adenocarcinoma adjusted for smoking (OR = 3.0; 95% CI: 1.2, 7.7, based on 10 exposed cases), although there was no information on the type of engine. Other case–control studies have suggested associations between other histological subtypes and diesel exposure (Olsson et al. 2011; Pintos et al. 2012; Villeneuve et al. 2011).

Several cohort studies have reported reduced risks for lung cancer among cotton textile workers in the United States, United Kingdom, and Shanghai, China, particularly those most exposed to endotoxins (Astrakianakis et al. 2007; Lundin and Checkoway 2009). In farming, a few studies have reported inverse associations with lung cancer, especially among farmers exposed to farm animals. In the AHS cohort, inverse associations were reported between lung cancer incidence and contact with poultry and large numbers of livestock, compared with farmers who did not raise these animals, after adjustment for smoking (Beane Freeman et al. 2012). Mastrangelo et al. (1996) reported an inverse relationship with duration of employment in cattle farming, compared with the general population. In a nested case–control analysis on lung cancer mortality among cattle farmers, they reported an inverse association with number of cattle, after adjustment for smoking (Mastrangelo et al. 2005). Those decreased risks were suspected to be linked to a high exposure to endotoxins, whose antitumor properties have been demonstrated, but the underlying mechanisms are unclear. Several mechanisms have been proposed, with a focus on complex interactions between the innate and adaptative immune systems (Lundin and Checkoway 2009). We found that the association with driving diesel tractors was greater among farmers who did not have exposure to animals or stored grain for adenocarcinoma than among those who were exposed to these factors. This pattern of increased risk among those not exposed to animals or stored grain was similar among users of combines and other crop harvesters, which were likely to be diesel-powered at the time period of enrollment, whereas inverse associations were observed for use of combines among farmers exposed to animals or stored grain. Among spouses, after adjustment for use of gasoline tractors, which was inversely related to lung cancer, positive associations with use of diesel tractors were found for both lung cancer and adenocarcinomas among spouses not exposed to animals, whereas decreased risks were observed among those exposed to animals. These findings should be interpreted with caution considering small numbers of cases (< 10) when analyses were stratified by exposure to animals or stored grain and performed by histological subtypes.

The prospective design of this study is a strength. In this unique agricultural cohort, we were able to provide discrimination throughout the year between no exposure, low (less than daily) exposure, intermediate (daily in one season) exposure, and high exposure (daily in both seasons) in farmers. Given our analyses, we did not see evidence of confounding by several factors, including smoking and pesticides.

Certain limitations should be acknowledged. Although this is a large cohort and we had reasonable numbers of exposed lung cancer cases for some analyses, numbers were small for some subgroups. We could not perform additional analyses by state or by type of tractor (with or without an enclosed cab). We were also limited in our analyses of the spouses because the highest exposure category collected was at least monthly in at least one season, limiting our ability to compare results directly with the farmers and to detect risks at higher exposures. We also had no duration of use or quantitative exposure data; nor did we have information on lifetime history of exposure to various engine exhausts. Data on size, age, and exhaust location of farm equipment were not collected. More stringent emissions standards for non-road engines have been established in the United States only since 2001; thus the impact of this on the AHS population is likely limited (Scheepers and Vermeulen 2012). Among farmers, 72% of drivers of diesel tractors used gasoline tractors and almost all drivers of gasoline tractors (95%) used diesel tractors. This overlap limited our ability to evaluate the risk associated with use of gasoline tractors independently of exposure to diesel exhaust. However, in models including information on use of gasoline and diesel tractors, the association with use of diesel tractors did not change substantially, whereas associations with use of gasoline tractors were attenuated. We used activities with animals or stored grain at the time of enrollment as a proxy for possible exposure to endotoxins, which were associated with a decreased risk of lung cancer (Beane Freeman et al. 2012; Mastrangelo et al. 2005). We were unable to consider historical and quantitative exposure to endotoxins. However, farmers were likely to have been exposed for several decades—nearly 75% of farmers had worked or lived on a farm for > 30 years at the time of enrollment. We cannot totally rule out that this effect modification was found by chance, as a result of the multiplicity of tests performed, or due to residual confounding.

## Conclusions

This study is one of the few to examine associations between the use of dieselized farm equipment and the risk of lung cancer among farmers and the first, to our knowledge, to evaluate the heterogeneity in risk by possible endotoxin exposure. We found an increased risk of adenocarcinomas, associated with daily use of diesel tractors among those not exposed to endotoxin-related activities (animals, stored grain); our results were, however, based on a few exposed cases. Although our results suggest that endotoxin may reduce the risk of diesel-induced lung cancer, future studies would benefit from more detailed assessment of exposure to engine exhaust and direct measurement of endotoxin exposures.

## Supplemental Material

(335 KB) PDF
