# Supplementary material for: Use of Dieselized Farm Equipment and Incident Lung Cancer: Findings from the Agricultural Health Study Cohort
Source: Environ Health Perspect. 2015 Oct 9;124(5):611–8. doi: 10.1289/ehp.1409238 (PMC4858397; doi:10.1289/ehp.1409238)
Supplement: (335 KB) PDF [file ehp.1409238.s001.acco.pdf]

Environ Health Perspect
DOI: 10.1289/ehp.1409238

Note to readers with disabilities: *EHP* strives to ensure that all journal content is accessible to all readers. However, some figures and Supplemental Material published in *EHP* articles may not conform to 508 standards due to the complexity of the information being presented. If you need assistance accessing journal content, please contact ehp508@niehs.nih.gov. Our staff will work with you to assess and meet your accessibility needs within 3 working days.

Supplemental Material

Use of Dieselized Farm Equipment and Incident Lung Cancer: Findings from the Agricultural Health Study Cohort

Séverine Tual, Debra T. Silverman, Stella Koutros, Aaron Blair, Dale P. Sandler, Pierre Lebailly, Gabriella Andreotti, Jane A. Hoppin, and Laura E. Beane Freeman

Table of Contents

Table S1. Associations between driving farm equipment and the overall lung cancer risk, mutually adjusted for the use of diesel and gasoline tractors, among farmers and female spouses of farmers, Agricultural Health Study

Table S2. Associations between driving farm equipment and the overall lung cancer risk, mutually adjusted for the use of diesel and gasoline tractors, by exposure to endotoxin-related activities, among farmers and female spouses of farmers, Agricultural Health Study

Table S3. Associations between driving farm equipment and lung cancer histological subtypes, mutually adjusted for the use of diesel and gasoline tractors, among farmers and female spouses of farmers, Agricultural Health Study

Table S4. Associations between driving farm equipment and risk for lung adenocarcinoma, mutually adjusted for the use of diesel and gasoline tractors, by exposure to endotoxin-related activities, among farmers and female spouses of farmers, Agricultural Health Study

Table S1. Associations between driving farm equipment and the overall lung cancer risk, mutually adjusted for the use of diesel and gasoline tractors, among farmers and female spouses of farmers, Agricultural Health Study

Exposure		Farmers		Spouses of farmers	
		Cases[a] n=281 N	RR[b] (95% CI)	Cases[a] n=160 N	RR[b] (95% CI)
Diesel tractors[c]					
	No	32	1.00	120	1.00
	≥Monthly ≥ 1 season	25	1.20 (0.77, 2.03)	33	0.88 (0.54, 1.43)
	≥Weekly ≥ 1 season	116	1.07 (0.71, 1.62)		
	Daily in one season	51	1.20 (0.74, 1.95)		
	Daily in both seasons	34	1.38 (0.81, 2.37)		
	p for trend[d]		0.27		
Gasoline tractors[c]					
	No	90	1.00	128	1.00
	≥Monthly ≥ 1 season	55	0.87 (0.62, 1.23)	25	0.67 (0.40, 1.15)
	≥Weekly ≥ 1 season	89	1.10 (0.81, 1.49)		
	Daily in one season	11	0.94 (0.49, 1.79)		
	Daily in both seasons	13	1.12 (0.61, 2.08)		
	p for trend[d]		0.58		
Combines or other types of crop harvesters[c]					
	No	80	1.00	141	1.00
	1-10 days	53	1.02 (0.70,1.49)	10	0.76 (0.38, 1.51)
	11-30 days	84	0.98 (0.67,1.43)		
	≥ 31 days	30	0.74 (0.46,1.18)		
	p for trend[d]		0.27		
Trucks[c]					
	No	82	1.00	106	1.00
	≥Monthly ≥ 1 season	40	0.83 (0.56, 1.22)	46	0.97 (0.66, 1.41)
	≥Weekly ≥ 1 season	67	0.87 (0.62, 1.22)		
	Daily in one season	14	0.95 (0.53, 1.71)		
	Daily in both seasons	49	0.73 (0.50, 1.08)		
	p for trend[d]		0.16		

[a] Case counts do not sum to total counts because of missing values for exposure variables
[b] For farmers, rate ratios were adjusted for age, pack-years (non-smokers, <20, 20-39, 40-59, ≥60, missing), state, race, level of education, current exposure to animals and stored grain (no, one exposure, both exposure), diesel tractor use (no, monthly, weekly, daily in one season, daily in both seasons) and gasoline tractor use (no, monthly, weekly, daily in one season, daily in both seasons); for spouses: rate ratios were adjusted for age, pack-years (non-smokers, <20, 20-39, ≥40, missing), level of education, exposure to farm animals in the year before enrollment, diesel tractor use (no, any) and gasoline tractor use (no, any)
[c] Test for trend, *p* value obtained by treating the categorical variable as ordinal

Table S2. Associations between driving farm equipment and the overall lung cancer risk, mutually adjusted for the use of diesel and gasoline tractors, by exposure to endotoxin-related activities, among farmers and female spouses of farmers, Agricultural Health Study

Population	Exposure		Nonexposed to endotoxin-related activities[a]		Exposed to endotoxin -related activities[a]		
			Cases[b] n	RR[c] (95% CI)	Cases[b] n	RR[c] (95% CI)	*p* for interaction[g]
Farmers	Diesel tractors	No/low[d]	70	1.00	103	1.00	0.87
		Intermediate[e]	10	0.99 (0.50, 1.97)	41	1.15 (0.79, 1.67)	
		High[f]	5	1.45 (0.55, 3.82)	29	1.24 (0.82, 1.88)	
	Gasoline tractors	No/low[d]	80	1.00	154	1.00	0.60
		Intermediate[e]/high[f]	5	1.27 (0.48, 3.38)	19	0.94 (0.58, 1.54)	
	Combines or other types of crop harvesters	0 day	45	1.00	35	1.00	0.64
		1-30 days	28	1.24 (0.75, 2.03)	109	0.91 (0.61, 1.36)	
		≥31 days	6	0.78 (0.32, 1.93)	24	0.68 (0.39, 1.18)	
	Trucks	No/low[d]	61	1.00	128	1.00	0.66
		Intermediate[e]	4	0.74 (0.26, 2.06)	10	1.27 (0.66, 2.44)	
		High[f]	16	0.78 (0.44, 1.37)	33	0.85 (0.57, 1.28)	
Spouses	Diesel tractors	No/<monthly	74	1.00	46	1.00	<0.01
		≥Monthly	19	1.81 (0.98, 3.34)	14	0.42 (0.21, 0.86)	
	Gasoline tractors	No/<monthly	82	1.00	46	1.00	0.37
		≥Monthly	11	0.53 (0.25, 1.14)	14	0.85 (0.42, 1.75)	
	Combines or other types of crop harvesters	0 day	89	1.00	52	1.00	0.20
		≥1 day	3	0.47 (0.14, 1.54)	7	1.20 (0.50, 2.88)	
	Trucks	No/<monthly	71	1.00	35	1.00	0.33
		≥Monthly	22	0.81 (0.48, 1.36)	24	1.18 (0.68, 2.05)	

[a] Endotoxin-related activities were defined by current exposure to animals or stored grain in farmers and exposure to animals in the year before enrollment in spouses
[b] Case counts do not sum to total counts because of missing values for exposure variables
[c] For farmers : rate ratios were adjusted for age, cigarette pack-years (non-smokers, <20, 20-39, 40-59, ≥60, missing), state, race, level of education, diesel tractor use (no/low, intermediate, high) and gasoline tractor use (no/low, intermediate/high); for spouses : rate ratios were adjusted for age, pack-years (non-smokers, <20, 20-39, ≥40, missing), level of education, diesel tractor use (no, any) and gasoline tractor use (no, any)
[d] No or low exposure was defined by a driving < daily in one season
[e] Intermediate exposure : daily driving in one season
[f] High exposure : daily driving in both seasons
[g] *p* for interaction was obtained from the likelihood ratio test by adding cross-product terms between each category of exposure and the variable reflecting potential exposure to endotoxins

Table S3. Associations between driving farm equipment and lung cancer histological subtypes, mutually adjusted for the use of diesel and gasoline tractors, among farmers and female spouses of farmers, Agricultural Health Study

		Farmers						Spouses	
		Adenocarcinoma		Squamous cell carcinoma		Small cell carcinoma		Adenocarcinoma	
Population	Exposure	Cases[a] n=78 n	RR[b] (95% CI)	Cases[a] n=75 n	RR[c] (95% CI)	Cases[a] n=50 n	RR[c] (95% CI)	Cases[a] n=69 n	RR[d] (95% CI)
Diesel tractors									
	No	7	1.00	6	1.00	12	1.00	46	1.00
	≥Monthly ≥ 1 season	7	1.58 (0.55, 4.57)	8	2.00 (0.68, 5.85)			17	1.01 (0.49, 2.09)
	≥Weekly ≥ 1 season	31	1.43 (0.60, 3.40)	34	1.57 (0.63, 3.90)	23	0.96 (0.45, 2.04)		
	Daily in one season	19	2.32 (0.90, 5.96)	12	1.41 (0.49, 4.03)	5	0.47 (0.15, 1.46)		
	Daily in both seasons	11	2.97 (1.06, 8.36)	7	1.31 (0.40, 4.28)	7	0.92 (0.32, 2.66)		
	*p f*or trend[e]		0.02		0.89		0.54		
Gasoline tractors									
	No	24	1.00	25	1.00	20	1.00	49	1.00
	≥Monthly ≥ 1 season	16	0.95 (0.50, 1.80)	15	0.82 (0.43, 1.58)	8	0.59 (0.26, 1.36)	14	0.94 (0.44, 2.00)
	≥Weekly ≥ 1 season	27	1.28 (0.72, 2.27)	23	0.95 (0.52, 1.71)	11	0.64 (0.30, 1.36)		
	Daily ≥1 season	8	1.25 (0.54, 2.89)	4	0.61 (0.20, 1.82)	8	1.92 (0.77, 4.77)		
	p for trend[e]		0.38		0.55		0.81		
Combines or other types of crop harvesters									
	Never	26	1.00	17	1.00	14	1.00	57	1.00
	1-10 days	11	0.67 (0.31, 1.42)	22	1.81 (0.90, 3.64)	9	1.04 (0.41, 2.59)	6	1.01 (0.40, 2.52)
	11-30 days	28	0.97 (0.50, 1.90)	18	0.90 (0.41, 1.97)	16	1.39 (0.57, 3.39)		
	≥31 days	10	0.75 (0.33, 1.72)	8	0.89 (0.35, 2.31)	5	0.85 (0.27, 2.67)		
	p for trend[e]		0.71		0.45		0.94		
Trucks									
	No	24	1.00	23	1.00	16	1.00	45	1.00
	≥Monthly ≥ 1 season	9	0.65 (0.30, 1.42)	11	0.75 (0.36, 1.56)	9	0.91 (0.40, 2.07)	17	0.74 (0.40, 1.36)
	≥Weekly ≥ 1 season	22	1.00 (0.55, 1.83)	15	0.64 (0.32, 1.25)	11	0.65 (0.29, 1.47)		
	Daily ≥ 1 season	18	0.77 (0.39, 1.51)	16	0.71 (0.35, 1.43)	10	0.45 (0.19, 1.10)		
	p for trend[e]		0.64		0.25		0.07		

[a] Case counts do not sum to total case counts because of missing values for exposure variables

[b] Adjusted for age (<55,55-59,60-64,65-69,>70), pack-years (non smokers, <20, 20-39,40-59, ≥60, missing), state, race, level of education (less than high school, high school, above high school, unknown), current exposure to animals and stored grain (no, one exposure, both exposure), diesel tractor use (no, monthly, weekly, daily in one season, daily in both seasons) and gasoline tractor use (no, monthly, weekly, daily in at least one season)

[c] Adjusted for age (<55,55-59,60-64,65-69,>70), pack-years (non smokers or <20, 20-39,40-59, ≥60, missing), state, race, level of education (less than high school, high school, above high school, unknown), current exposure to animals and stored grain (No, one exposure, both exposure), diesel tractor use (no, monthly, weekly, daily in one season, daily in both seasons) and gasoline tractor use (no, monthly, weekly, daily in at least one season)

[d] Adjusted for age (<55,55-59,60-64,65-69,>70), pack-years (non smokers, <20, 20-39, ≥40, missing), level of education (high school or less, above high school, unknown), exposure to farm animals in the year before enrollment, diesel tractor use (no, any) and gasoline tractor use (no, any)

[e] Test for trend, *p* value obtained by treating the categorical variable as ordinal

Table S4. Associations between driving farm equipment and risk for lung adenocarcinoma, mutually adjusted for the use of diesel and gasoline tractors, by exposure to endotoxin-related activities, among farmers and female spouses of farmers, Agricultural Health Study

Population	Exposure		Nonexposed to endotoxin -related activities[a]		Exposed to endotoxin -related activities[a]		
			Cases[b] N	RR[c] (95% CI)	Cases[b] N	RR[c] (95% CI)	*p* for interaction[h]
Farmers	Diesel tractors	No/low[d]	18	1.00	27	1.00	0.24
		Intermediate[e]	3	1.33 (0.38, 4.74)	16	1.72 (0.91, 3.27)	
		High[f]	4	4.09 (1.18, 14.18)	7	1.23 (0.52, 2.88)	
	Gasoline tractors	No/low[d]	23	1.00	45	1.00	0.35
		Intermediate[e]/high[f]	3	1.79 (0.45, 7.16)	5	0.79 (0.31, 2.04)	
	Combines or other types of crop harvesters	0 day	11	1.00	14	1.00	0.09
		1-30 days	9	1.56 (0.62, 3.91)	30	0.56 (0.28, 1.12)	
		≥31 days	4	1.97 (0.54, 7.16)	6	0.37 (0.14, 1.02)	
	Trucks	No/low[d]	16	1.00	39	1.00	0.74
		Intermediate[e]/high[f]	7	0.97 (0.39, 2.45)	11	0.80 (0.39, 1.62)	
Spouses[g]	Diesel tractors	No/<monthly	29	1.00	17	1.00	0.03
		≥Monthly	9	2.16 (0.88, 5.33)	8	0.47 (0.17, 1.32)	
	Gasoline tractors	No/<monthly	33	1.00	16	1.00	0.16
		≥Monthly	5	0.54 (0.17, 1.66)	9	1.57 (0.58, 4.27)	
	Trucks	No/<monthly	30	1.00	15	1.00	0.21
		≥Monthly	8	0.78 (0.43, 1.41)	9	0.25 (0.03, 1.96)	

[a] Endotoxin-related activities were defined by current exposure to animals or stored grain in farmers and current exposure to animals in spouses
[b] Case counts do not sum to total counts because of missing values for exposure variables
[c] For farmers : Rate ratios were adjusted for age, pack-years (non smokers, <20, 20-39,40-59, ≥60, missing), state, race, level of education(less than high school, high school, above high school, unknown), diesel tractor use (no/low, intermediate, high) and gasoline tractor use (no/low, intermediate/high); for spouses : rate ratios were adjusted for age, pack-years (non-smokers, <20, 20-39, ≥40, missing), level of education (high school or less, above high school, unknown), diesel tractor use (no, any) and gasoline tractor use (no, any)
[d] No or low exposure was defined by a driving < daily in one season
[e] Intermediate exposure : daily driving in one season
[f] High exposure : daily driving in both seasons
[h] *p* for interaction was obtained from the likelihood ratio test by adding cross-product terms between each category of exposure and the variable reflecting potential exposure to endotoxins
[g] Associations were not shown for use of combines or other types of crop harvesters for spouses due to too few exposed cases (1 case among spouses not exposed to farm animals, 5 cases among those exposed to farm animals)
